# Median Nerve to Biceps Nerve Transfer to Restore Elbow Flexion in Obstetric Brachial Plexus Palsy

**DOI:** 10.1155/2014/854084

**Published:** 2014-01-09

**Authors:** M. M. Al-Qattan, T. M. Al-Kharfy

**Affiliations:** ^1^Department of Surgery, King Saud University, P.O. Box 18097, Riyadh 11415, Saudi Arabia; ^2^Medical College, King Saud University, P.O. Box 18097, Riyadh 11415, Saudi Arabia; ^3^Department of Pediatrics, King Saud University, P.O. Box 18097, Riyadh 11415, Saudi Arabia

## Abstract

Median nerve to biceps nerve transfer in the arm has been reported only in adults. The following paper reports on 10 cases of this transfer in obstetric brachial plexus palsy. All patients had upper palsy (ERb's or extended ERb's palsy) and presented to the author late (13–19 months of age) with poor or no recovery of elbow flexion. Following the nerve transfer, nine children recovered elbow flexion (a score of 6 in one child and a score of 7 in eight children by the Toronto scale). The remaining child did not recover elbow flexion.

## 1. Introduction

ERB's palsy (C5 and C6 root injury) and extended ERb's palsy (C5, C6, and C7 root injury) are the two most common types of obstetric brachial plexus injuries and they also carry better prognosis for spontaneous recovery than the total palsy [1]. Infants with ERb's/extended ERb's palsy who do not show good spontaneous recovery undergo exploration of the brachial plexus and reconstruction of the injured roots in infancy (usually between 3 and 9 months of age) using a combination of intra- and extraplexus neurotization in the neck [2]. In the presence of root avulsion, the priority in these two types of obstetric palsy is given to the biceps (usually through intraplexus nerve grafting to the anterior division of the upper trunk) and to shoulder stability/external rotation (usually through extraplexus spinal accessory nerve to suprascapular nerve transfer) [3].

Children with ERb's/extended ERb's palsy presenting late (1-2 years of age) with poor or no recovery of the biceps usually undergo distal nerve transfer. One of the most commonly used distal nerve transfers is known as the Oberlin nerve transfer [4]: a fascicle of the ulnar nerve (supplying the flexor carpi ulnaris muscle) is cut and sutured end-to-end to the biceps nerve in the upper arm. Oberlin used this transfer in adults and the first use of such a transfer in obstetric palsy was by Al-Qattan in 2002 [5]. Later, many other authors reported Oberlin nerve transfer in infants with obstetric palsy [6–10].

Another common distal nerve transfer to reinnervate the biceps is the median nerve to biceps nerve transfer in the arm. This transfer has only been used in adults [11–13], and our review revealed no studies investigating the results of this transfer in obstetric palsy.

The current paper reports on the results of ten cases of obstetric brachial plexus palsy in which the median nerve to biceps nerve transfer was used to reinnervate the biceps.

## 2. Patients and Methods

This work was approved by the Research Committee at the Department of Surgery, King Saud University. A retrospective review of the data sheets of our obstetric brachial plexus clinic [14] was done to identify cases of median nerve to biceps nerve transfers. The following data were collected: age, sex, type of palsy, preoperative motor assessment of elbow flexion, age at the time of surgery, operative procedure, complications, and outcome (postoperative motor assessment of elbow flexion) at the time of final follow-up. We use the Toronto motor assessment scale [2] as shown in Table 1.

## 3. Patient Selection

At our obstetric brachial plexus center, the indication for primary brachial plexus surgery (in patients presenting early, soon after birth) is lack of active elbow flexion against gravity at 4 months of age. Patients presenting late (4 months to one year of age) with poor or no recovery of elbow flexion will undergo biceps neurotization at the neck level (using intraplexus neurotization of the anterior division of the upper trunk). If the patient is older than one year of age, biceps neurotization is done at the arm level using either a fascicle from the median or ulnar nerve. Our cut-off for biceps neurotization at the arm level is 20 months and older children will undergo either pedicled muscle transfer or free functional muscle transfer to reconstruct elbow flexion.

Another important point is the definition of “poor” recovery of elbow flexion in birth palsy patients presenting late. At our center, a muscle grade of 4 or less (see Table 1) is an absolute indication for surgery. Patients with a muscle grade of 5 will be assessed for triceps cocontraction. If the biceps muscle grade does not improve to 6 or 7 by treating the cocontraction with Botulinum toxin, these children will also undergo biceps neurotization. Finally, children with a grade of 6 elbow flexion do not undergo biceps neurotization.

## 4. Preoperative Evaluation

All patients are assessed by history and clinical examination documented in our own data sheets [14]. No clinical sensory assessment is done because it is difficult to test sensation in these young children. Clinical motor assessment is done using the Toronto grading system (Table 1) rather than the standard MRC grading system because the former is much easier to apply to infants and young children. We do not obtain electrical studies routinely in birth palsy patients because they are very difficult to interpret and the results will not affect our decision to operate specially in patients who present late with poor or no recovery of the biceps. Similarly, patients presenting late will not undergo MRI, not only because the results will not affect the decision for the distal nerve transfer, but also because the MRI requires a general anesthesia in these children. It is important to note that none of the patients in the current series have had initial primary brachial plexus reconstruction earlier.

## 5. Surgical Technique of Median Nerve to Biceps Nerve Transfer

A longitudinal incision is made along the bicipital sulcus of the medial aspect of the upper arm. The musculocutaneous nerve is identified and followed up distally to identify the biceps branch. The biceps nerve is dissected from the main trunk of the musculocutaneous nerve proximally (for 1-2 cm) and divided. The median nerve is then identified and neurolysis is performed opposite to the area of the identified biceps nerve. Using a nerve stimulator, the motor fascicle innervating the flexor carpi radialis is identified. The donor fascicle is divided distally and flipped towards the biceps nerve. Nerve coaptation is done with fibrin glue and not sutures. The incision is closed with no drains.

## 6. Postoperative Care and Rehabilitation

In the operating room, an arm sling is applied. The sling is removed after 3 weeks. Once the sling is removed, passive exercises of the shoulder and elbow joints are done daily at home by the parents as well as weekly at the physiotherapy department. Any contractures are treated by appropriate splinting. Following early reinnervation with early joint motion (Grades 2–4, Table 1), strengthening of the biceps with “gravity eliminated” is performed. Once a Grade 5 is reached, strengthening “against gravity” is performed. Passive exercises are continued to prevent joint contractures.

## 7. Results

All patients in the current series presented late, and surgery was done within three weeks of presentation to our clinic. A summary of the data of our cases that underwent median nerve to biceps nerve is shown in Table 2. Age at the time of presentation ranged from 13 to 19 months. There were 7 cases of ERb's palsy (C5 and C6 injury) and 3 cases of extended ERb's palsy (C5, C6, and C7 injury). The preoperative motor assessment of elbow flexion ranged from 0 to 2. At final follow-up (1-2 years after surgery), all seven ERb's cases obtained a score of 7 out of 7 for elbow flexion. Two cases with extended ERb's had a score of 6 and 7. The only failure was in a case of extended ERb's, who presented very late (19 months of age).

None of the patients had anesthetic or wound complications. Finally, no clinically detectable donor site deficits were seen in the hand in any of the patients.

## 8. Discussion

The current series is the first series of median nerve to biceps nerve transfer in obstetric palsy. We obtained a good result in 9 of the 10 cases. It is interesting to note that the only failure was seen in the child who presented the latest (19 months of age) and the same child had extended ERb's palsy. In adults, the results of distal nerve transfer for biceps reinnervation are also known to be worse in very late presentations and in extended ERb's palsy compared to relatively early presentations and ERb's palsy [4, 13].

Patient selection for nerve transfer in birth palsy varies from one center to another. Belzberg et al. [15] conducted a multinational survey of peripheral nerve surgeons and found that the most significant areas of disagreement included the timing and indications for surgical intervention in birth-related palsy. A long period of muscle denervation has many adverse effects such as muscle fiber atrophy, neuromuscular junction changes, increased fibrosis in the distal nerve, and a decline in Schwann cells in the distal nerve [16]. At our center, the age range for distal nerve transfer for the biceps is between 12 and 20 months of age. The cut-off of 20 months would probably be too late in adults with ERb's palsy who frequently have root avulsion and hence there is complete denervation of the biceps. In ERb's birth palsy upper root avulsion is rare and hence the biceps may not be completely denervated (some axons regenerate through the neuroma of ruptured roots). Furthermore, axonal regeneration is quicker in children compared to adults. Finally, electrical stimulation of denervated muscles is routinely performed to our infants with birth palsy and this factor may also add to the better chance of recovery of muscle after delayed innervation in our patient population [17].

Despite all the factors that improve the outcome of delayed neurotization of the biceps in children with birth palsy, a long period of denervation is still associated with a higher risk of failure. Our only failure (case number 9, Table 2) was seen in the child who presented at 19 months of age. It is important to sit with the parents and discuss the risk of failure and obtain an informed consent. Most parents give the consent despite the risk of failure because we inform them that distal nerve transfer does not result in any detectable donor nerve morbidity and that failure of the distal nerve transfer will not have any adverse effects on further management using muscle transfers.

In adults, it is well known that the double nerve transfer for elbow flexion reconstruction (i.e., ulnar nerve to biceps nerve and median nerve to brachialis nerve) gives better results than single nerve transfer (i.e., either ulnar nerve or median nerve to biceps nerve transfer only without reinnervation of the brachialis). In children, however, the result of Oberlin single nerve transfer alone has yielded consistently good results [5, 7–10] and our study shows that the same results can be obtained with median nerve single nerve transfer for biceps reinnervation in obstetric palsy.

There are many options of biceps reinnervation in obstetric palsy such as the transfer of the intercostal or the pectoral nerves [18–20]. However, the ulnar and median nerve transfers are generally preferred in late presentations since the donor fascicle is closer to the biceps nerve, hence providing a shorter period of reinnervation. In fact, the use of median nerve may gain popularity in very late presenting cases because the median nerve is closer to the biceps than the ulnar nerve.

## Figures and Tables

**Table 1:** The Toronto scale of motor assessment.

Scale	Description	Testing done with
0	Nil	Gravity eliminated
1	Muscle contraction, no motion	Gravity eliminated
2	Joint motion ≤ 1/2 range	Gravity eliminated
3	Joint motion > 1/2 range	Gravity eliminated
4	Full motion	Gravity eliminated
5	Joint motion ≤ 1/2 range	Against gravity
6	Joint motion > 1/2 range	Against gravity
7	Full motion	Against gravity

**Table 2:** Data of the 10 cases who underwent median nerve to biceps nerve transfer.

Number	Age/sex	Type of palsy	Preoperative motor assessment of elbow flexion	Final follow-up after surgery	Postoperative motor assessment of elbow flexion at final follow-up
1	14 months/M	ERb's	0	1 year	7
2	13 months/F	ERb's	2	2 years	7
3	15 months/F	ERb's	0	2 years	7
4	14 months/M	ERb's	2	1.5 years	7
5	16 months/F	ERb's	0	1 year	7
6	13 months/M	ERb's	2	2 years	7
7	13 months/M	ERb's	2	2 years	7
8	16 months/F	Extended ERb's	0	1.5 years	6
9	19 months/F	Extended ERb's	0	1 year	0
10	14 months/M	Extended ERb's	0	1 year	7

Assessment according to the Toronto scale shown in Table 1.

M: male, F: female.
